# Supplementary material for: Effect of Obesity on Aquaporin5 Expression in Human Placental and Uterus Tissues
Source: J Clin Med. 2024 Jul 31;13(15):4490. doi: 10.3390/jcm13154490 (PMC11312882; doi:10.3390/jcm13154490)
Supplement: Supplementary file 1 [file jcm-13-04490-s001.zip › jcm-3116945-supplementary.pdf]

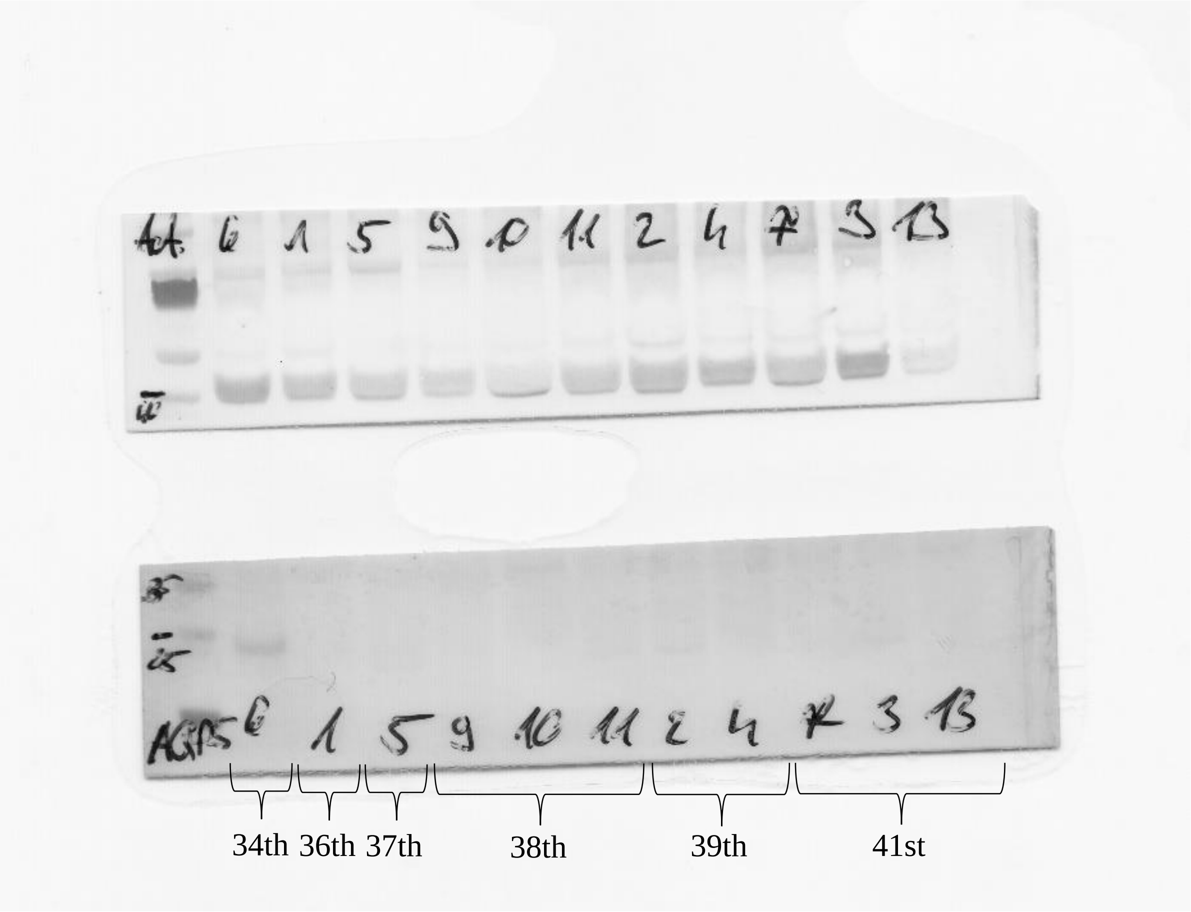

Supplement 1. Changes in the AQP5 protein expression during various weeks of pregnancy in human uterine samples (Fig. 3)

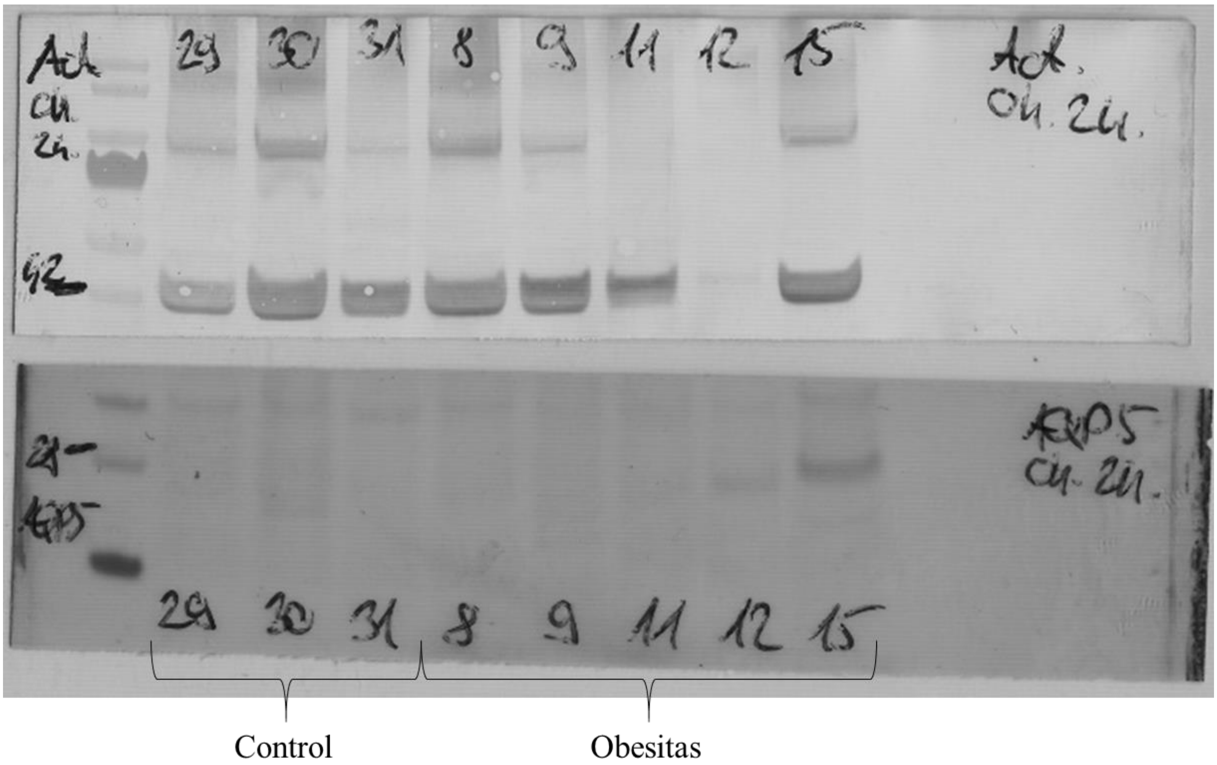

Supplement 2. Changes in the AQP5 protein expression in the uterine tissue samples collected from obese and control (non-obese) women (Fig. 5)

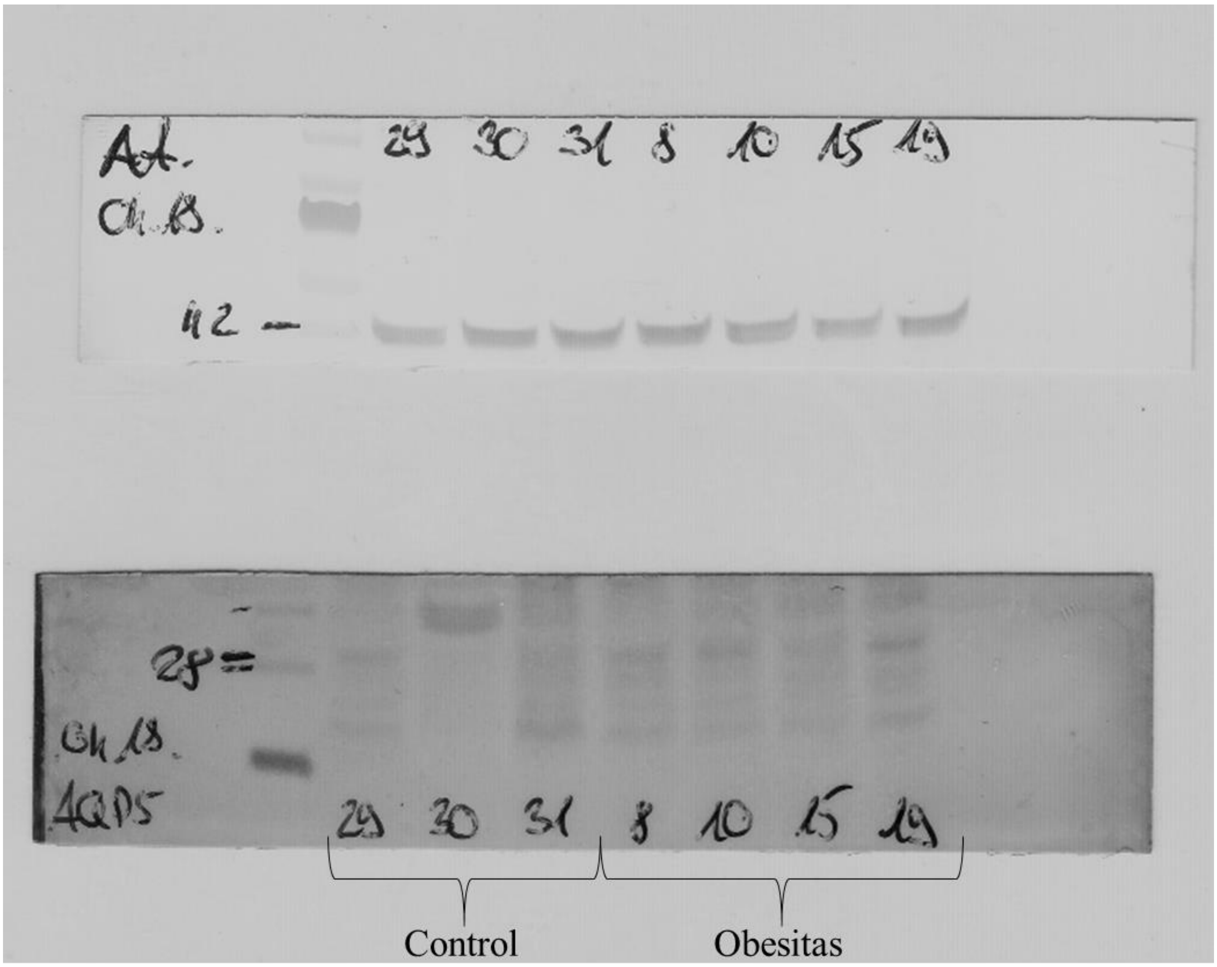

Supplement 3. Changes in the AQP5 protein expression in the placental tissue samples collected from obese and control (non-obese) women (Fig. 6).
